# Spartin activates atrophin-1-interacting protein 4 (AIP4) E3 ubiquitin ligase and promotes ubiquitination of adipophilin on lipid droplets

**DOI:** 10.1186/1741-7007-8-72

**Published:** 2010-05-26

**Authors:** Christopher Hooper, Swamy S Puttamadappa, Zak Loring, Alexander Shekhtman, Joanna C Bakowska

**Affiliations:** 1 Department of Molecular Pharmacology and Therapeutics, Loyola Chicago University, Maywood, IL, USA; 2 Department of Chemistry, SUNY, Albany, NY, USA

## Abstract

**Background:**

Spartin protein is involved in degradation of epidermal growth factor receptor and turnover of lipid droplets and a lack of expression of this protein is responsible for hereditary spastic paraplegia type 20 (SPG20). Spartin is a multifunctional protein that associates with many cellular organelles, including lipid droplets. Recent studies showed that spartin interacts with E3 ubiquitin ligases that belong to the neural precursor cell-expressed developmentally downregulated gene (Nedd4) family, including atrophin-1-interacting protein 4 (AIP4/ITCH). However, the biological importance of the spartin-AIP4 interaction remains unknown.

**Results:**

In this study, we show that spartin is not a substrate for AIP4 activity and that spartin's binding to AIP4 significantly increases self-ubiquitination of this E3 ligase, indicating that spartin disrupts the AIP4 autoinhibitory intramolecular interaction. Correspondingly, spartin has a seven times higher binding affinity to the WW region of AIP4 than the binding of the WW region has to the catalytic homologues of the E6-associated protein C-terminus (HECT) domain, as measured by enzyme-linked immunosorbent assay. We also show that spartin recruits AIP4 to lipid droplets and promotes ubiquitination of lipid droplet-associated protein, adipophilin, which regulates turnover of lipid droplets.

**Conclusions:**

Our findings demonstrate that spartin acts as an adaptor protein that activates and recruits AIP4 E3 ubiquitin ligase to lipid droplets and by this means regulates the level of ubiquitination of adipophilin and potentially other lipid-associated proteins. We propose that this is one of the mechanisms by which spartin regulates lipid droplet turnover and might contribute to the pathology of SPG20.

## Background

The hereditary spastic paraplegias (HSPs) are inherited neurological disorders characterized by progressive spasticity and muscle weakness in the lower limbs [1]. Troyer syndrome is an autosomal recessive HSP caused by a frameshift mutation in the spartin gene (*SPG20*) [2], resulting in a loss of expression of the mutated protein [3].

Spartin harbors a microtubule-interacting and trafficking (MIT) domain and a plant-senescence domain at its N-terminus and C-terminus, respectively [4]. The latter domain was suggested to be responsible for spartin's association with lipid droplets in cells incubated with oleic acid [5]. Spartin has a multifunctional role in cells as evidenced by its association with membranes of several cellular organelles [5,6] and its interaction with many binding partners [7]. Thus far is it known that spartin is important in the trafficking of epidermal growth factor receptors [6,8] and in the turnover of lipid droplets [5]. Lipid droplets contain a neutral lipid core surrounded by a monolayer of polar lipids. The membrane layer of the lipid droplet has two major groups of proteins: enzymes important in lipid metabolism and the perilipin, adipophilin, and 47-kDa tail interacting protein (PAT) family of peripheral membrane proteins involved in stabilizing lipid droplets [9].

Spartin is known to be ubiquitinated and interacts with several E3 ubiquitin ligases that harbor a homologous to E6-associated protein C-terminus (HECT) domain. These ligases belong to the neural precursor cell-expressed developmentally downregulated gene (Nedd4) family and include atrophin-1-interacting protein 4 (AIP4) [8,10] and atrophin-1-interacting protein 5, also known as the WW domain-containing E3 ubiquitin protein ligase 1 (AIP5/WWP1) [5,8]. Although previous studies have shown that AIP5 is responsible for ubiquitination of spartin and removes spartin from the lipid droplets [5], a recent report demonstrated that depletion of AIP4 and AIP5 had no effect on spartin's ubiquitination [8]. Furthermore, the importance of the interaction between spartin and E3 ubiquitin ligases with the HECT domain remains unknown.

In this study, we provide evidence that spartin is not ubiquitinated by AIP4; rather, spartin acts as an adaptor protein that, through binding to AIP4, increases the enzymatic activity of this E3 ligase by releasing AIP4 from its autoinhibited state. We also demonstrate that spartin recruits AIP4 to lipid droplets and is a necessary adaptor protein for polyubiquitination of adipophilin on lipid droplets. This might be one of the mechanisms by which spartin regulates the turnover of lipid droplets.

## Results

### Ubiquitination levels of spartin are not altered by AIP4

Previous studies showed that spartin interacts with AIP2 (also known as WW domain-containing protein 2 (WWP2)), AIP4, and AIP5 through its PPAY motif [5,8]. However, there is a discrepancy between previous studies: one found that spartin is a substrate of AIP4 and AIP5 E3 ligases [5], but another found that spartin is not a substrate of those ligases [8]. To resolve this discrepancy, we used an *in vivo *ubiquitination assay to investigate the ubiquitination status of spartin in cells overexpressing ubiquitin together with either wild-type or enzymatically inactive AIP4. Specifically, we examined the conjugation of hemagglutinin (HA)-tagged ubiquitin into Myc-spartin that had been immunoprecipitated from HeLa cells cotransfected with ubiquitin and spartin together with either Flag-AIP4 or Flag-AIP4C830A.

**Figure 1:**
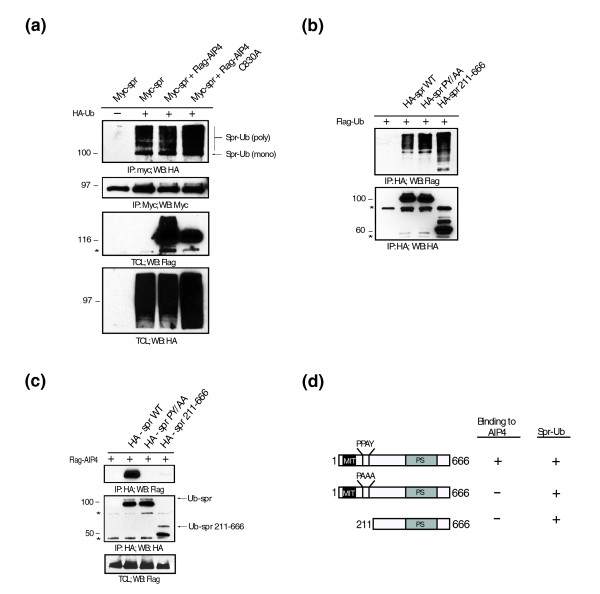
**Atrophin-1-interacting protein 4 (AIP4) does not ubiquitinate spartin**. **(a) **HeLa cells were transfected with Myc-wild-type spartin together with an empty vector (lane 1), hemagglutinin (HA)-ubiquitin (lane 2), HA-ubiquitin and Flag-AIP4 (lane 3), or HA-ubiquitin and Flag-AIP4C830A (lane 4). TCL = total cell lysates. WB = western blot. An asterisk (*) indicates crossreactive proteins. Sizes of protein standards are indicated to the left in kDa. **(b) **Immunoprecipitates of HA-wild-type, mutant PY/AA, or 211-666 fragments of spartin and Flag-ubiquitin analyzed by immunoblotting with anti-Flag (upper panel) or anti-HA (lower panel) antibodies. **(c) **HeLa cells were transfected with Flag-AIP4 alone or together with HA-wild-type spartin, HA-spartin PY/AA, or HA-spartin 211-666 fragment. Cell lysates were immunoprecipitated (IP) with anti-HA antibodies and immunoblotted with anti-Flag (upper panel) or anti-HA (lower panel) antibodies. An asterisk (*) and kDa are indicated as in A. **(d) **Schematic diagram of spartin constructs and their status of ubiquitination and binding to AIP4.

Before immunoprecipitation, cell lysates were boiled in sodium dodecyl sulfate (SDS) to eliminate proteins that could coprecipitate with spartin. Immunoblots showed that, in the presence of wild-type AIP4 or catalytically inactive AIP4C830A, the ubiquitination levels of overexpressed spartin were similar (Figure 1a). As we previously shown [6], spartin was monoubiquitinated and polyubiquitinated when overexpressed with exogenous ubiquitin. However, the polyubiquitination of spartin does not result in its increased degradation by proteasomes (data not shown).

The interaction between spartin and AIP4 or AIP5 is mediated by spartin's PPAY motif. When the PPAY motif within spartin was mutated to PAAA, it abrogated the interaction with AIP5 and AIP4 ([5,8] and this study). One report found that this mutation resulted in the loss of ubiquitination of spartin [5], but Edwards and colleagues [8] reported that this mutation had no effect on the levels of spartin's ubiquitination. To resolve these contradictory results, we further examined the ubiquitination levels of spartin after preventing its binding to AIP4 by mutating the PPAY motif or using the fragment of spartin depleted of its N-terminus containing the PPAY motif. We overexpressed HA-wild-type spartin, HA-mutant spartin PY/AA (in which PPAY motif was mutated to PAAA) or HA-tagged spartin fragment 211-666 (which lacks the PPAY motif) together with Flag-ubiquitin in HeLa cells. As shown in Figure 1b, overexpressed spartin PY/AA and the spartin fragment (211-666) were both monoubiquitinated and polyubiquitinated to the same extent as the wild-type spartin. Neither overexpressed spartin PY/AA nor the spartin fragment (211-666) interacted with AIP4 (Figure 1c). We summarized the association of different spartin constructs with AIP4 and their ubiquitination status in Figure 1d. Overall, our results indicate that the levels of spartin's ubiquitination are not affected by its association with AIP4.

### Spartin recruits AIP4 to endogenous lipid droplets

**Figure 2:**
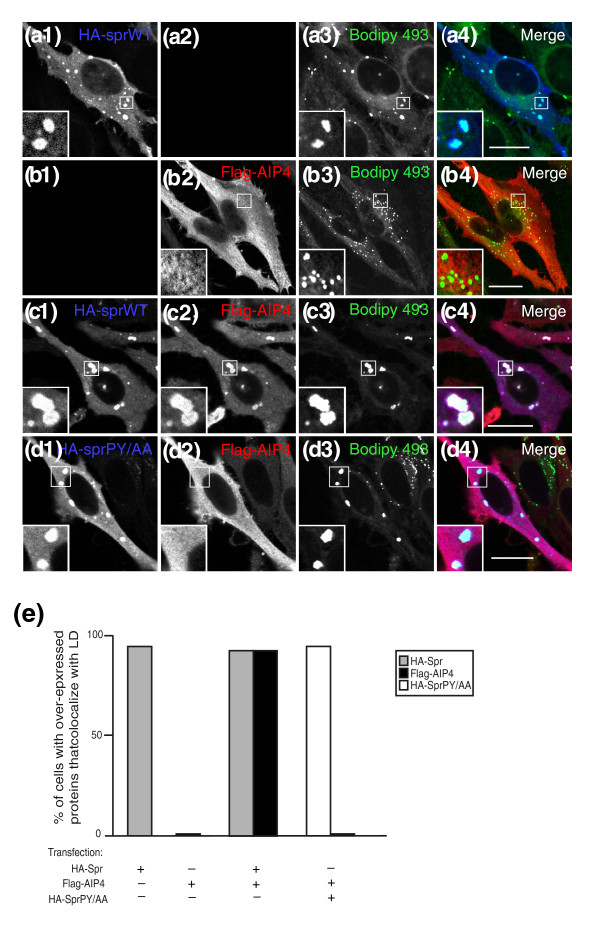
**Spartin recruits atrophin-1-interacting protein 4 (AIP4) to lipid droplets**. **(a) **HeLa cells were transfected with hemagglutinin (HA)-spartin and immunostained for HA in blue (A1) and stained for lipid droplets with Bodipy 493/503 in green (A3). Merged image is shown in A4. The boxed areas are enlarged in the insets. **(b) **HeLa cells were transfected with Flag-AIP4 and immunostained for Flag in red (B2) and stained for lipid droplets with Bodipy 493/503 in green (B3). Merged image is shown in B4. The boxed areas are enlarged in the insets. **(c) **HeLa cells were cotransfected with HA-spartin and Flag-AIP4 and immunostained for HA in blue (C1), for Flag in red (C2), and for lipid droplets with Bodipy 493/503 in green (C3). Merged image is shown in C4. The boxed areas are enlarged in the insets. **(d) **HeLa cells were cotransfected with HA-spartin PY/AA and Flag-AIP4 and immunostained for HA in blue (D1), for Flag in red (D2), and for lipid droplets with Bodipy 493/503 in green (D3). Merged image is shown in D4. The boxed areas are enlarged in the insets. Bars = 20 μm. **(e) **Colocalization of indicated expressed proteins with lipid droplets was scored for 30 cells in each of 3 independent experiments. Transfected vectors are indicated with '+' below each column.

**Figure 3:**
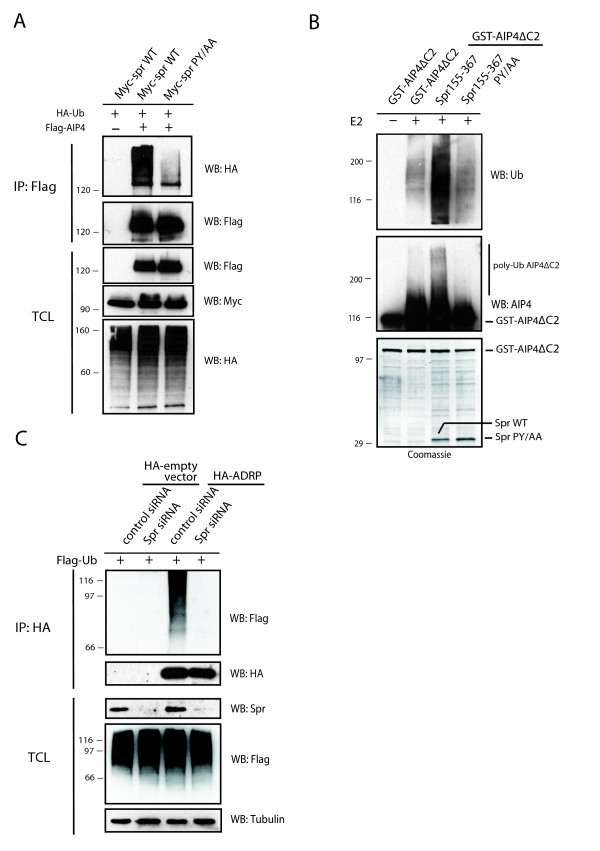
**Spartin increases activity of atrophin-1-interacting protein 4 (AIP4) and promotes ubiquitination of adipophilin**. **(a) **HeLa cells were cotransfected with Myc-wild-type spartin together with hemagglutinin (HA)-ubiquitin and an empty vector (lane 1), or Flag-AIP4 (lane 2), or Myc-spartin PY/AA with HA-ubiquitin and Flag-AIP4 (lane 3). Cell lysates were boiled in sodium dodecyl sulfate (SDS) before immunoprecipitation (IP) with anti-Flag antibodies. Levels of HA-ubiquitin and Flag-AIP4 in immunoprecipitates and total cell lysates (TCL) were analyzed by immunoblotting. **(b) **Levels of *in vitro *ubiquitination assay of glutathione S-transferase (GST)-AIP4ΔC2 alone (lane 2), with spartin155-367 (lane 3), or mutant spartin 155-367PY/AA (lane 4) determined by immunoblot with anti-ubiquitin antibodies (upper panel). All reactions (except lane 1) were carried out in the presence of E1 ubiquitin-activating enzyme, E2 ubiquitin-conjugating enzyme, ATP, and ubiquitin. Coomassie blue stained gel (bottom panel). **(c) ***In vivo *ubiquitination assay of HA-adipophilin, also known as adipose differentiation-related protein (ADRP) in HeLa cells transfected with either HA-empty vector or HA-ADRP, and control or spartin small interfering RNA (siRNA) and Flag-ubiquitin and treated with 300 μM of oleic acid. Cell lysates were immunoprecipitated with anti-HA antibodies and immunoblotted with anti-Flag or anti-HA antibodies.

**Figure 4:**
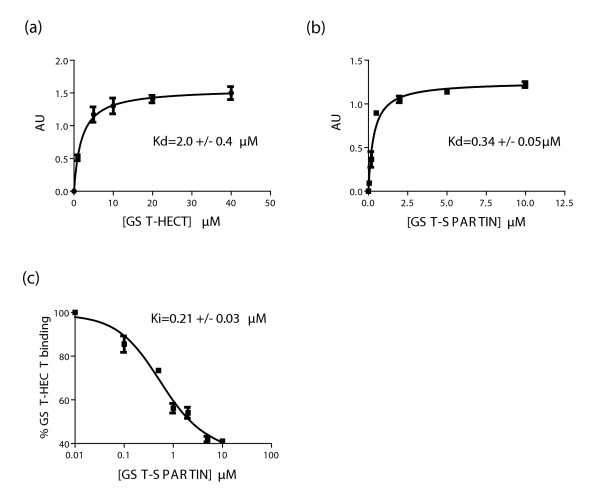
**Binding of proline-rich region (PRR)/WWI-IV to the homologues of the E6-associated protein C-terminus (HECT) domain of atrophin-1-interacting protein 4 (AIP4) and wild-type (WT) spartin**. **(a) **Binding isotherm of the glutathione S-transferase (GST)-HECT protein to PRR/WWI-IV. AU = arbitrary units. **(b) **Binding isotherm of the GST-WT spartin 155-367 protein to PRR/WWI-IV. **(c) **Inhibition of GST-HECT binding to PRR/WWI-IV by WT spartin. All experiments were repeated three times. Data are shown as mean ± SD with an R^2 ^≥ 0.95.

Although spartin interacts with AIP4, it is still ubiquitinated in the presence of an enzymatically inactive mutant of AIP4, suggesting that spartin is not a substrate of AIP4. Those data prompted us to investigate whether spartin acts as an adaptor protein that alters the localization of AIP4 in the cell. We used immunofluorescence to examine the subcellular localization of overexpressed AIP4 alone or together with the wild-type spartin or mutant spartin PY/AA, which does not bind to AIP4. We found that HA-spartin (Figure 2a, 1) was localized in the cytoplasm and associated with endogenous lipid droplets (Figure 2a, 4) in nearly all transfected cells (Figure 2e). In contrast, overexpressed Flag-AIP4 (Figure 2b, 2) did not associate with lipid droplets in any examined cells (Figure 2b, 4). However, when HA-spartin and Flag-AIP4 were cotransfected, Flag-AIP4 was recruited to endogenous lipid droplets by HA-wild-type spartin in nearly all cotransfected cells (Figure 2c). Cells coexpressing HA-mutant spartin PY/AA and Flag-AIP4 showed diffuse staining of AIP4 (Figure 2d, 2) but no colocalization with the mutant spartin (Figure 2d, 1) in lipid droplets (Figure 2d, 3) in any cotransfected cells. However, HA-mutant spartin PY/AA (Figure 2d, 1) associated with lipid droplets (Figure 2d, 4) similarly to HA-wild-type spartin (Figure 2c, 1 and 2d, 4).

We also found that HA-wild-type spartin but not mutant spartin PY/AA sequestered AIP2 to the lipid droplets (Additional file 1). These results reveal that spartin is an adaptor protein that recruits AIP2 and AIP4 to the lipid droplets.

### Spartin increases the activity of AIP4 *in vivo *and *in vitro*

To further investigate the role of spartin as an adaptor protein in binding with AIP4, we examined whether, in addition to recruiting AIP4 to lipid droplets, spartin also enhances its enzymatic activity. It has been shown that, because of the presence of the catalytic HECT domain, the level of self-ubiquitination of AIP4 is a good measure of its enzymatic activity [11,12].

We examined the levels of AIP4 ubiquitination in HeLa cells in which Flag-AIP4 was cotransfected with HA-ubiquitin and either Myc-wild-type spartin or Myc-mutant spartin PY/AA, which does not bind to AIP4. In order to dissociate all proteins that could potentially coprecipitate with AIP4, we boiled cell lysates in SDS then decreased its concentration in lysate buffer. We performed immunoprecipitation with anti-Flag antibodies and immunoblotted the membrane with anti-HA antibodies to detect ubiquitinated Flag-AIP4. As shown in Figure 3a, the level of Flag-AIP4 ubiquitination (represented by a high molecular weight smear of HA-ubiquitin) was significantly higher in cells cotransfected with wild-type spartin than in cells cotransfected with mutant spartin that does not bind AIP4.

Furthermore, we examined whether binding of wild-type spartin to AIP4 *in vitro *also results in increased self-ubiquitination of AIP4. We investigated the levels of self-ubiquitination of the glutathione S-transferase (GST)-AIP4ΔC2 protein in the absence of spartin or in the presence of spartin fragments 155-367 or 155-367 PY/AA. We used these spartin fragments because they are easily expressed in bacteria. We used AIP4 lacking the C2 domain because this protein contains all necessary domains for its autoinhibition and is well expressed in bacteria [11]. As shown in Figure 3b (lane 2), in the absence of spartin, AIP4ΔC2 was self-ubiquitinated at low levels in the presence of all necessary components for *in vitro *ubiquitination. When spartin 155-367 was added to the reaction, self-ubiquitination of AIP4ΔC2 increased significantly as detected by anti-ubiquitin antibodies (Figure 3b, lane 3, top panel). In the presence of spartin, a high molecular weight smear was also detected when we used anti-ITCH antibodies (Figure 3b, lane 3, second panel from the top). In contrast, in the presence of mutant spartin 155-367 PY/AA, AIP4ΔC2 was ubiquitinated at the same low levels as in the absence of spartin (Figure 3b, lanes 4 and 2, respectively). The results from *in vitro *and *in vivo *ubiquitination experiments demonstrate that spartin's binding to AIP4 significantly increases the catalytic activity of this E3 ligase.

### Spartin promotes polyubiquitination of adipophilin on lipid droplets

Although spartin is not ubiquitinated by AIP4, by recruiting AIP4 to lipid droplets and increasing its activity, spartin might place AIP4 in the vicinity of its substrate(s). One of those substrates might be adipophilin, also known as adipose differentiation-related protein (ADRP), which has been shown to be polyubiquitinated [13]. To examine the levels of ubiquitination of adipophilin in the absence of AIP4, AIP5, and AIP2, we did not attempt to knock down AIP4, AIP5, and AIP2 simultaneously in cells because depletion of two of these E3 ubiquitin ligases results in cell death [14]. Using immunofluorescence, we determined that spartin colocalizes with HA-adipophilin on lipid droplets (Additional file 2). Thus, we tested whether spartin-mediated recruitment of Nedd4 E3 ubiquitin ligases to lipid droplets might be responsible for the polyubiquitination of adipophilin. We found that spartin cells treated with small interfering RNA (siRNA) showed significantly less polyubiquitination of adipophilin compared with control siRNA-treated cells (Figure 3c). This suggests that spartin-mediated recruitment of Nedd4 E3 ligases to lipid droplets might be responsible for ubiquitination of adipophilin in HeLa cells.

### Binding of spartin to AIP4 disrupts the proline-rich region (PRR)/WW-HECT complex

Other researchers have suggested that the enzymatic activity of AIP4 is autoinhibited by the intramolecular interaction of its HECT domain with the region between the proline-rich region and the WWIV domain [11,12]. Furthermore, we showed that spartin-mediated binding to AIP4 increases its self-ubiquitination, implying that spartin releases AIP4 from its autoinhibited state. To assess the inhibitory interactions of AIP4 directly, we purified the PRR/WWI-IV region and the HECT domain of AIP4 and used the intermolecular PRR/WW-HECT complex to mimic the autoinhibited state of AIP4; then we examined whether spartin's binding to AIP4 disrupts this complex. We sought to quantitatively measure the binding affinity between the PRR/WWI-IV region and the HECT domain of AIP4.

By using a capture enzyme-linked immunosorbent assay (ELISA), we detected saturable binding between the proline-rich region (PRR)/WWI-IV region and the HECT domain; the dissociation constant was 2 ± 0.4 μM (Figure 4a). We also observed saturable binding between spartin and the PRR/WWI-IV region. The calculated dissociation constant of this interaction was 0.34 ± 0.05 μM (Figure 4b). These results indicate that spartin has a seven times higher affinity of binding to the PRR/WWI-IV region than the binding of the PRR/WWI-IV region has to the HECT domain. We assessed whether the binding of spartin to AIP4 would disrupt the PRR/WW-HECT complex, which would release AIP4 from its autoinhibited state. As shown in Figure 4c, wild-type spartin inhibited the intermolecular interaction between HECT and the PRR/WWI-IV fragment in a dose-dependent manner, with a half maximal inhibitory concentration (IC_50_) of 0.56 ± 0.08 μM and an inhibition constant of 0.21 ± 0.03 μM. In contrast, mutant spartin PA/PY failed to inhibit the HECT-PRR/WWI-IV complex. Thus, spartin increases enzymatic activity of AIP4 indirectly by disrupting its PRR/WW-HECT complex releasing AIP4 from it autoinhibited state.

## Discussion

Hereditary spastic paraplegias, such as Troyer syndrome, are neurodegenerative diseases characterized by progressive weakness and spasticity in the lower limbs [1]. A nucleotide deletion (1110delA) in *spartin *(*SPG20*) causes autosomal recessive Troyer syndrome [2]. Previously, we demonstrated that this mutation results in a complete loss of spartin and postulated that Troyer syndrome has a loss-of-function disease mechanism [3].

Recently, it has been shown that spartin is involved in turnover of lipid droplets by an unknown mechanism [5]. These findings suggested that there is a link between lipid droplet turnover and the pathology of Troyer syndrome. In the present studies, we investigated a molecular mechanism for spartin-dependent regulation of lipid droplet turnover. This novel pathway, which we call SPAA, involves spartin, the E3 ubiquitin ligase AIP4, and a major lipid droplet protein, adipophilin.

Our study provides molecular and biochemical evidence that spartin is not ubiquitinated by AIP4 but acts as an adaptor protein for this E3 ubiquitin ligase. We determined that spartin markedly increases self-ubiquitination of AIP4 through PPAY-mediated binding and recruits it to the lipid droplets. We also demonstrated that spartin promotes ubiquitination of adipophilin, a lipid-associated protein that is known to affect triacylglycerol turnover [15].

**Figure 5:**
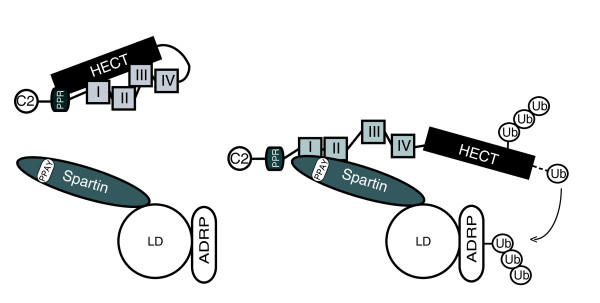
**Model of spartin's role in the activation and recruitment of atrophin-1-interacting protein 4 (AIP4) to lipid droplets and subsequent adipophilin ubiquitination**. AIP4 in the absence of binding to spartin is in an autoinhibited conformation (left panel). Our heuristic model predicts that spartin, via its PPAY motif, recruits AIP4 to lipid droplets and alters AIP4 conformation, which leads to its increased enzymatic activity. The ubiquitination of resident proteins present on lipid droplets, such as adipophilin, is facilitated by the spartin-mediated recruitment and activation of AIP4 (right panel). I, II, III, IV = WW-I, WW-II, WW-III, and WW-IV domains; ADRP = adipophilin (also known as adipose differentiation-related protein); C2 = calcium binding domain; HECT = homologues of the E6-associated protein C-terminal domain; LD = lipid droplet; PPAY = PPAY motif of spartin; PRR = proline-rich domain; Ub = ubiquitin.

The catalytic activity of AIP4, similar to other E3 ligases in the Nedd4 family [16], is regulated by intramolecular and intermolecular interactions. The intramolecular interactions lead to the autoinhibition of the catalytic activity of many members of the Nedd4 family E3 ligases [11,16]. It has been shown that autoinhibition of AIP4 is due to the binding between its HECT domain and WW domains [12] or its HECT domain and a sequence between the PRR and the WW domains [11]. The current model that explains the increase of AIP4 activity involves disrupting intramolecular binding within AIP4, which releases the HECT domain from autoinhibition. We tested this model and demonstrated quantitatively that PPAY-mediated binding of spartin to AIP4 disrupts the interaction between the HECT domain and the PRR/WW region of AIP4. The binding affinity of the PRR/WW region for spartin (K_d _= 0.3 μM) is about seven times stronger than the binding affinity for the HECT domain (K_d _= 2 μM) *in vitro*. These data strongly support our model in which a tight interaction between spartin and the WW-I and WW-II domains of AIP4 (Additional file 3) disrupts a weaker intramolecular interaction, which leads to increased catalytic activity of this E3 ligase (Figure 5). Spartin interacts with other E3 ligases belonging to the Nedd4 family, including AIP2 and AIP5 [5,8], which are known to have a propensity for autoinhibition [11]. Therefore, we anticipate that spartin might also instigate the enzymatic activity of these other E3 ubiquitin ligases and will investigate them in future studies.

We have shown that spartin, through its PPAY motif, sequesters AIP2 and AIP4 to endogenous lipid droplets. This finding contrasts with that of previous studies that showed decreased accumulation of YFP-spartin on lipid droplets (stimulated with 500 μM of oleic acid) by overexpressed Cherry-AIP5 [5]. One possible explanation for this discrepancy is that the induction of lipid droplets with a high dose of oleic acid and the large fluorescence tags fused to both spartin and AIP5 significantly altered the dynamics of those proteins.

We demonstrated that spartin facilitates ubiquitination of adipophilin, a lipid droplet-associated protein. It has been shown that adipophilin is polyubiquitinated and then degraded by a proteasome-dependent pathway [13]. Ubiquitination is one of the mechanisms by which the stability of adipophilin is regulated [13], and its level of ubiquitination seems to be regulated by spartin-mediated recruitment of Nedd4 E3 ubiquitin ligases to lipid droplets (Figure 5). Because adipophilin decreases triacylglycerol turnover [15,17], we propose that spartin affects lipid droplet turnover ([5], and this study) (Additional file 4) by regulating the level of ubiquitination of adipophilin and perhaps other lipid droplet-associated proteins. We call this molecular mechanism the SPAA pathway for lipid droplet turnover.

Impaired lipid droplet metabolism might be one of the mechanisms in the pathogenesis of Troyer syndrome. Malfunction of proteins involved in lipid maintenance is considered to be a cause of some neurological disorders [18]. For example, an autosomal-dominant hereditary spastic paraplegia, HSP17, which is characterized by muscle weakness in upper limbs and spasticity of lower limbs, is caused by a mutation in the Berardinelli-Seip congenital lipodystrophy 2 (*BSCL2*) gene encoding seipin [19]. Seipin has been shown to maintain the physiological morphology and size of lipid droplets in yeast [20]. A large number of lipid droplets in atrophic α motor neurons have been reported in hereditary porcine neuronal degeneration [21]. Now, based on the SPAA pathway for lipid droplet turnover, we can begin unraveling the specific biological role of spartin in Troyer syndrome.

## Conclusions

In summary, spartin is not a substrate for AIP4 E3 ubiquitin ligase. Instead, spartin acts as a classic adaptor protein that recruits AIP4 to lipid droplets and increases its enzymatic activity. In addition, spartin acts as a linker for ubiquitination of adipophilin on lipid droplets, likely by sequestering Nedd 4 E3 ubiquitin ligases and ubiquitination machinery. These might be one of the mechanisms by which spartin regulates lipid droplet turnover and which may contribute to the pathophysiology of SPG20.

## Methods

### Antibodies and reagents

The following antibodies were used: rabbit polyclonal anti-hemagglutinin (HA; Abcam, Cambridge, MA, USA), mouse monoclonal anti-HA (12CA5; Covance, Princeton, NJ, USA), rabbit polyclonal anti-Myc, mouse monoclonal anti-Flag conjugated to horseradish peroxidase (HRP) (clone M2; Sigma, St. Louis, MO, USA), mouse monoclonal anti-ITCH (BD Biosciences, San Jose, CA, USA), mouse monoclonal anti-ubiquitin P4D1 (Cell Signaling, Danvers, MA, USA), and rabbit polyclonal anti-glutathione S-transferase (GST) (Santa Cruz, Santa Cruz, CA, USA). The rabbit anti-spartin antibody was used as described previously [22]. The anti-mouse and anti-rabbit antibodies conjugated to HRP were from Thermo Fischer Scientific (Waltham, MA, USA). Secondary goat anti-mouse and anti-rabbit Alexa Fluor 568-conjugated or 633-conjugated antibodies were from Invitrogen (Carlsbad, CA, USA)

### Mammalian expression vectors

The full-length human spartin cDNA in the pGW1-HA and pGW1-Myc expression vectors have been described previously [6]. Flag-AIP4 in a pCMV-10 vector was a gift from Adriano Marchese (Loyola Chicago University, Maywood, IL, USA). To generate the pGW1-HA spartin 211-666 construct, residues 211-666 of human spartin were amplified by PCR using *PfuTurbo *(Agilent Technologies, Santa Clara, CA, USA) and cloned in frame into an *Eco*RI site of the pGW1-HA vector. To generate spartin PY172,174AA and Flag-AIP4C830A, site-directed mutagenesis was applied using the QuickChange protocol (Agilent Technologies). We refer to mutant spartin PY172,174AA as spartin PY/AA. HA-adipophilin was obtained from GeneCopoeia (Rockville, MD, USA). The Flag-AIP2 vector was a gift from Richard Longnecker (Northwestern University, Evanston, IL, USA).

### Bacterial expression vectors

The pGEX-4T2 with each WW domain I, II, III, IV and pGEX-4T2 with HECT domain were gifts from Adriano Marchese (Loyola Chicago University). A vector expressing GST-AIP4ΔC2 was a gift from Annie Angers (University of Montreal, Montreal, Canada). The pGEX6p-1-PRR-WWI-IV (155-486) vector was generated by PCR using full-length AIP4 as a template. To generate pGEX6p-1-wild-type spartin 155-367 or pGEX6p-1-spartin 155-367 PY172,174AA, human spartin was amplified by PCR using either wild-type or mutant spartin cDNA as a template.

### Cell culture and transfections

HeLa cells were maintained in Dulbecco's modified Eagle's medium (Invitrogen) supplemented with 10% fetal bovine serum (Gemini BioProducts, West Sacramento, CA, USA). The transfections with plasmids or plasmids together with control siRNA or spartin siRNA [6] were performed using the TransIT-HeLaMONSTER transfection kit (Mirus, Madison, WI, USA) and Lipofectamine 2000 (Invitrogen), respectively.

### Immunofluorescence

HeLa cells were grown on glass cover slips, transfected, and processed as described previously [23]. Cover slips were mounted with ProLong Antifade reagent (Invitrogen) and imaged using a Zeiss LSM-510 confocal microscope (Zeiss, Maple Grove, MN) with a 63 × 1.4 NA Plan Apochromat oil immersion objective at 1024 × 1024 resolution. The images were processed with Adobe Photoshop 7.0 software (Adobe, San Jose, CA, USA).

### Image analysis

For each specified immunofluorescence condition, 30 cells that showed overexpressed proteins were analyzed at random, and colocalization was assessed with line scans using Zeiss software. A total of 90 cells were analyzed in 3 independent experiments. The data are represented as the percentage of cells that show colocalization of immunostained, overexpressed proteins with lipid droplets.

### *In vitro *ubiquitination

The assays were conducted in reaction buffer with reagents that were described previously [11] and with 1-2 μg of GST-AIP4ΔC2 alone or together with spartin 155-367 wild-type or spartin 155-367 PY172,174AA proteins, both of which were cleaved of their GST tag by using PreScission protease (GE Healthcare, Waukesha, WI, USA). The samples were incubated for 60 min at 37°C and analyzed by immunoblotting using anti-ubiquitin and anti-ITCH antibodies.

### *In vivo *ubiquitination

Transfected HeLa cells were washed twice with ice-cold phosphate-buffered saline (PBS) and lysed with buffer containing 20 mM Tris, 150 mM NaCl, 1% Triton X-100, 10 mM *N*-ethylmaleimide, and protease inhibitors. Cell lysates were denatured with 1% SDS, boiled for 5 min, and diluted 14 times with lysis buffer to lower the concentration of SDS before immunoprecipitation. For *in vitro *ubiquitination of HA-ADRP in cells treated with control or spartin siRNA, cells were treated with 300 μM of oleic acid overnight and then subsequently with MG132 for 3 h.

### ELISA

The following plasmids were used to express proteins in bacteria: pGEX-4T2-HECT, pGEX6p-1-PRR-WWI-IV, pGEX6p-1-wild-type (WT) spartin 155-367, and pGEX6p-1-spr 155-367 PY/AA. Proteins were purified as described previously [24]. We applied PreScission protease (GE Healthcare) to remove the N-terminal GST tag from GST-PRR/WWI-IV, GST-WT spartin 155-367, and GST-spartin 155-367 PY/AA. All proteins were dialyzed using PBS. ELISA microplates (96 well; BD Biosciences) were coated with 50 ng of PRR/WWI-IV per well in 10 mM phosphate buffer (pH 7.4) and incubated overnight at 4°C. After washes and blocking, serially diluted samples of GST-WT spartin 155-367, GST-spartin 155-367 PY/AA, or GST-HECT were added, and the plates were incubated for 2 h. The plates were washed with PBS and then incubated for 1 h with rabbit anti-GST. After the washes with PBS and 0.05% Tween 20, the plates were incubated with a 1:1,000 dilution of anti-rabbit HRP conjugated antibodies for 1 h. After washes with PBS, the color was developed with 3,3'5,5'-tetramethylbenzidine (Thermo Fisher Scientific) for 5 min, and the optical density was read at 630 nm. The assays were performed in triplicate. Prism 5 (Graphpad Software, La Jolla, CA, USA) was used to analyze binding data. For competition ELISA, 5 μM of GST-HECT was incubated with serially diluted WT spartin 155-367 or spartin 155-367 PY/AA in wells coated with 50 ng of PRR/WWI-IV. The amount of bound GST-HRP was detected using anti-GST antibodies.

### Analysis of ELISA data

The IC_50 _and K_d _were calculated by fitting the data points with either the one-site binding curve AU = AU_0 _× ((GST-protein)/((GST-protein) + K_d_) or the competitive binding curve %GST-HECT-binding = Min + (Max - Min)/(1 + 10^((WT spartin) - log(IC50)^), where AU (AU_0_) is the ELISA (maximal) absorption reading, (GST-protein) is either the GST-HECT or GST-WT spartin 155-367 concentration, (WT spartin) is the WT spartin 155-367 concentration, and Min(Max) is the minimal (maximal) percentage of GST-HECT binding. The inhibition constant, K_i_, was calculated by using the equation K_i _= IC_50_/(1 + (GST-HECT)/K_d_) [25] based on an IC_50_, concentration of bound GST-HECT, (GST-HECT) = 5 μM, and GST-HECT binding affinity for PRR/WWI-IV, K_d _= 2 μM.

## Authors' contributions

CH designed and performed all *in vitro *and *in vivo *ubiquitination assays and assisted in writing the manuscript. SSP designed and performed ELISA experiments. ZL performed the immunoprecipitation experiments. AS designed ELISA experiments and provided a critique of the manuscript. JCB conceived the studies, performed immunofluorescence experiments, and confocal analysis with CH's help, wrote the manuscript, and provided oversight of the project.

## Supplementary Material

Additional file 1 **Additional figure 1**. Spartin recruits atrophin-1-interacting protein 2 (AIP2) to lipid droplets. **(a) **HeLa cells were transfected with Flag-AIP2 and immunostained for Flag in red (A2), and lipid droplets were stained with Bodipy 493/503 in green (A3). Merged image is shown in A4. The boxed areas are enlarged in the insets. **(b) **HeLa cells were cotransfected with hemagglutinin (HA)-spartin and Flag-AIP2 and stained for HA in blue (B1), Flag in red (B2), and lipid droplets with Bodipy 493/503 in green (B3). Merged image is shown in B4. The boxed areas are enlarged in the insets **(c) **HeLa cells were cotransfected with HA-spartin PY/AA and Flag-AIP2 and stained for HA in blue (C1), Flag in red (C2), and for lipid droplets with Bodipy 493/503 in green (C3). Merged image is shown in C4. The boxed areas are enlarged in the insets. Bars = 20 μm. **(d) **Colocalization of indicated expressed proteins with lipid droplets was scored for 30 cells in each of 3 independent experiments. Transfected vectors are indicated with '+' below each column. Click here for file

Additional file 2 **Additional figure 2**. Spartin colocalizes with adipophilin (also known as adipose differentiation-related protein (ADRP)) in lipid droplets. **(a) **HeLa cells were cotransfected with Myc-spartin and hemagglutinin (HA)-ADRP and immunostained for Myc in blue (A1), HA in red (A2), and stained for lipid droplets with Bodipy 493/503 in green (A3). Merged image is shown in A4. **(b) **Colocalization of Myc-spartin and HA-ADRP with lipid droplets was scored for 30 cells in each of 3 independent experiments. Transfected vectors are indicated with '+' below each column. Click here for file

Additional file 3 **Additional figure 3**. Spartin interacts with the WW-I and WW-II domains of atrophin-1-interacting protein 4 (AIP4) *in vitro*. Upper panel: Lysates from HeLa cells were transfected with hemagglutinin (HA)-tagged wild-type spartin or HA-spartin PY/AA and were incubated with glutathione S-transferase (GST) alone or GST-WW I-IV of AIP4, or GST fused with each WW domain of AIP4. Bound proteins from the precipitation assay were immunoblotted with anti-HA antibodies. Lower panel: Coomassie blue stained gel. Click here for file

Additional file 4 **Additional figure 4**. Depletion of spartin increases the size and number of lipid droplets. **(a) **HeLa cells were treated with control (A1) or spartin small interfering RNA (siRNA) (A2) for 48 h and then incubated with 300 μM of oleic acid and stained for lipid droplets using Bodipy 493/503. **(b) **The bars show the average number of lipid droplets ± standard error in cells treated with control or spartin siRNA from 3 independent experiments using 30 cells each. An asterisk (*) represents significance at *P *< 0.01 calculated by Student t test. Click here for file
