# Supplementary figures and images for: Spartin activates atrophin-1-interacting protein 4 (AIP4) E3 ubiquitin ligase and promotes ubiquitination of adipophilin on lipid droplets
Source: BMC Biol. 2010 May 26;8:72. doi: 10.1186/1741-7007-8-72 (PMC2887783; doi:10.1186/1741-7007-8-72)

# Additional File1

## Figure 1

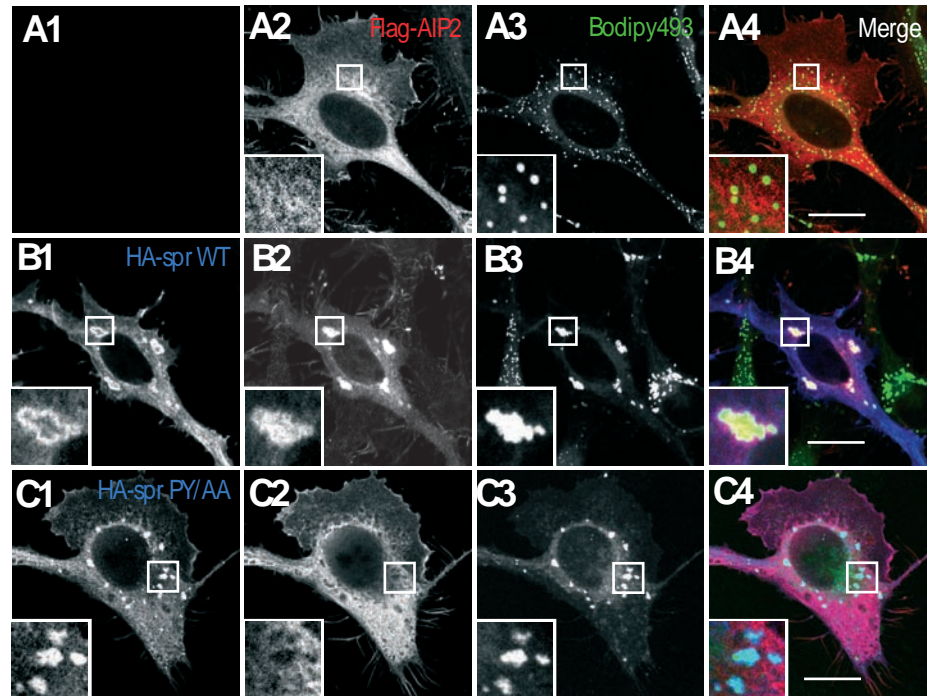

**D**

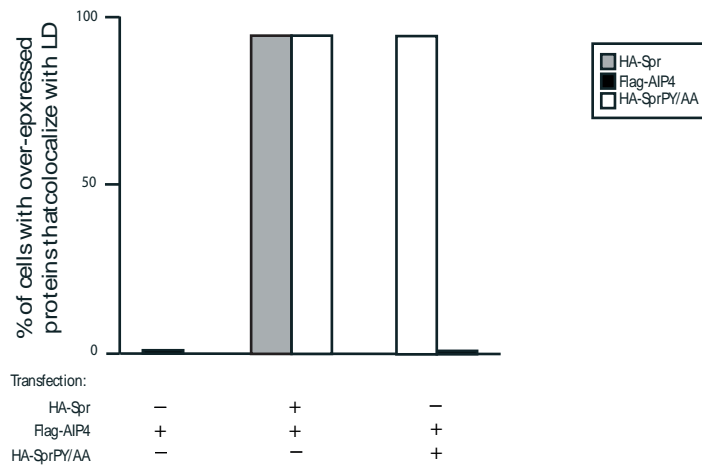

Supplement: Additional file 1 — Additional figure 1. Spartin recruits atrophin-1-interacting protein 2 (AIP2) to lipid droplets. (a) HeLa cells were transfected with Flag-AIP2 and immunostained for Flag in red (A2), and lipid droplets were stained with Bodipy 493/503 in green (A3). Merged image is shown in A4. The boxed areas are enlarged in the insets. (b) HeLa cells were cotransfected with hemagglutinin (HA)-spartin and Flag-AIP2 and stained for HA in blue (B1), Flag in red (B2), and lipid droplets with Bodipy 493/503 in green (B3). Merged image is shown in B4. The boxed areas are enlarged in the insets (c) HeLa cells were cotransfected with HA-spartin PY/AA and Flag-AIP2 and stained for HA in blue (C1), Flag in red (C2), and for lipid droplets with Bodipy 493/503 in green (C3). Merged image is shown in C4. The boxed areas are enlarged in the insets. Bars = 20 μm. (d) Colocalization of indicated expressed proteins with lipid droplets was scored for 30 cells in each of 3 independent experiments. Transfected vectors are indicated with '+' below each column. [file 1741-7007-8-72-S1.pdf]

# Additional File2

## Figure 2

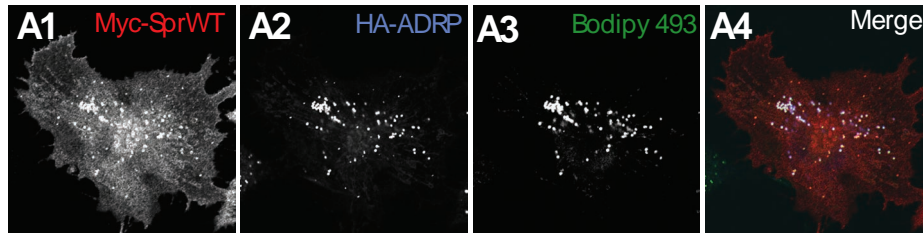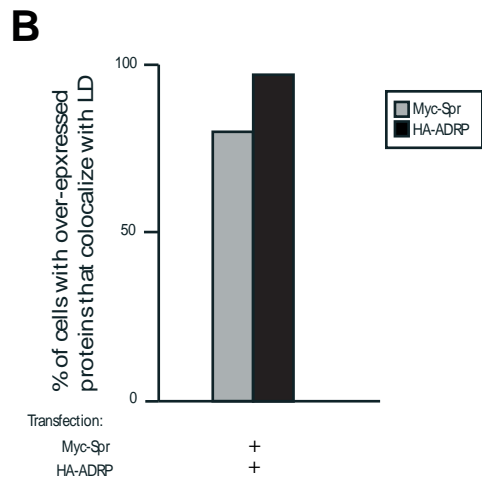

Supplement: Additional file 2 — Additional figure 2. Spartin colocalizes with adipophilin (also known as adipose differentiation-related protein (ADRP)) in lipid droplets. (a) HeLa cells were cotransfected with Myc-spartin and hemagglutinin (HA)-ADRP and immunostained for Myc in blue (A1), HA in red (A2), and stained for lipid droplets with Bodipy 493/503 in green (A3). Merged image is shown in A4. (b) Colocalization of Myc-spartin and HA-ADRP with lipid droplets was scored for 30 cells in each of 3 independent experiments. Transfected vectors are indicated with '+' below each column. [file 1741-7007-8-72-S2.pdf]

# Additional File3

## Figure 3

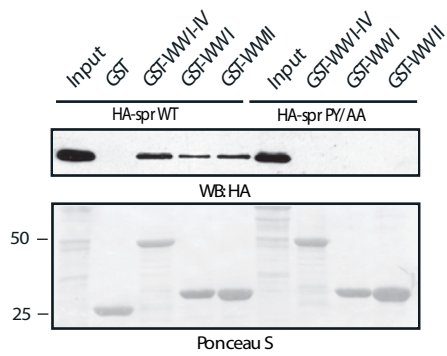

Supplement: Additional file 3 — Additional figure 3. Spartin interacts with the WW-I and WW-II domains of atrophin-1-interacting protein 4 (AIP4) in vitro. Upper panel: Lysates from HeLa cells were transfected with hemagglutinin (HA)-tagged wild-type spartin or HA-spartin PY/AA and were incubated with glutathione S-transferase (GST) alone or GST-WW I-IV of AIP4, or GST fused with each WW domain of AIP4. Bound proteins from the precipitation assay were immunoblotted with anti-HA antibodies. Lower panel: Coomassie blue stained gel. [file 1741-7007-8-72-S3.pdf]

## Additional File4

### Figure 4

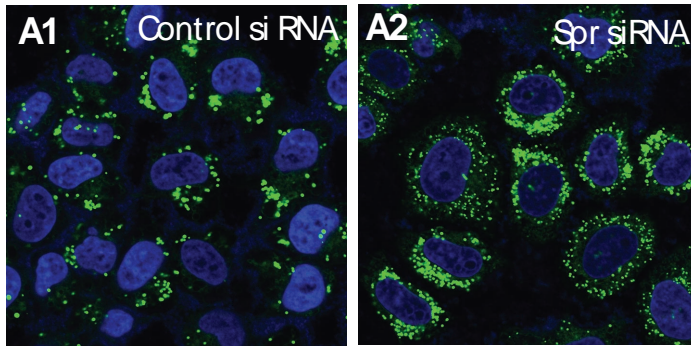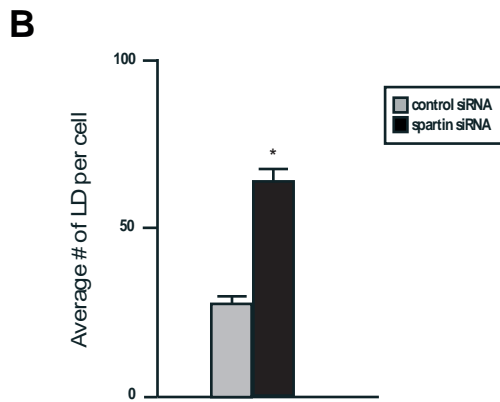

Supplement: Additional file 4 — Additional figure 4. Depletion of spartin increases the size and number of lipid droplets. (a) HeLa cells were treated with control (A1) or spartin small interfering RNA (siRNA) (A2) for 48 h and then incubated with 300 μM of oleic acid and stained for lipid droplets using Bodipy 493/503. (b) The bars show the average number of lipid droplets ± standard error in cells treated with control or spartin siRNA from 3 independent experiments using 30 cells each. An asterisk (*) represents significance at P < 0.01 calculated by Student t test. [file 1741-7007-8-72-S4.pdf]
